# Acupoint Catgut Embedding Improves Lipid Metabolism in Exercise-Induced Fatigue Rats via the PPAR Signaling Pathway

**DOI:** 10.3390/ani13040558

**Published:** 2023-02-05

**Authors:** Yue Song, Xiaoyu Shi, Zhenzhen Gao, Ran Li, Jiamin Tian, Xiaodong Cao, Bin Yang, Shihua Zhao, Ying Yang

**Affiliations:** 1 College of Veterinary, Inner Mongolia Agricultural University, Hohhot 010018, China; 2 Veterinary Research Institute, Inner Mongolia Academy of Agricultural & Animal Husbandry Sciences, Hohhot 010031, China; 3 School of Pharmacy, Inner Mongolia Medical University, Hohhot 010018, China

**Keywords:** transcriptome, metabolome, rat, exercise-induced fatigue

## Abstract

**Simple Summary:**

To improve the phenomenon of exercise-induced fatigue that often occurs during horse racing, we previously studied the improvement in exercise tolerance by acupoint catgut embedding preconditioning in an exercise-induced fatigue rat model. We found that acupoint catgut embedding pretreatment effectively improved animal exercise tolerance, but the mechanisms underlying these effects remain unclear. In this study, we explored the underlying mechanisms of this improvement by transcriptomic analysis. We showed that the PPAR signaling pathway was enriched through transcriptomic data analysis. Similarly, the mRNA expression levels of solute carrier family 27 member 2 (*Slc27a2*), fatty acid binding protein 1 (*Fabp1*), and apolipoprotein C3 (*Apoc3*) genes in the peroxisome proliferator-activated receptor (PPAR) pathway were decreased in the acupoint catgut group compared to the treadmill group. Further, to further explore the role of PPAR, we also detected the lipid metabolism index by using metabolomics. We found that acupoint embedding can correct the lipid metabolism index, i.e., free fatty acids (FFAs), arachidonic acid (AA), triglyceride (TG), etc., in the blood. Our study demonstrated that acupoint catgut embedding regulates the PPAR signaling pathway and further improves body fat metabolism. Our findings provide an important step to understanding how acupuncture catgut embedding improves exercise-induced fatigue (EF).

**Abstract:**

To improve the phenomenon of exercise-induced fatigue that often occurs during horse racing, we previously studied the improvement in exercise tolerance by acupoint catgut embedding preconditioning in an exercise-induced fatigue rat model. We found that acupoint catgut embedding pretreatment effectively improved animal exercise tolerance. Here, by combining transcriptomics and metabolomics, we aimed to explore the underlying mechanisms of this improvement. We used blood biochemical detection combined with ELISA to detect triglyceride (TG), total cholesterol (TC), lactate dehydrogenase (LDH), high-density lipoprotein (HDL), alanine transaminase (ALT), aspartate aminotransferase (AST), and glucose (GLU), arachidonic acid (AA), and free fatty acid (FFA) content and found that acupoint embedding can correct FFA, AA, TG, LDH, and AST in the blood. We used RT-qPCR to measure the expression of genes in tissue from the quadriceps femoris muscle. We found that solute carrier family 27 member 2 (*Slc27a2)*, fatty acid binding protein 1 (*Fabp1)*, apolipoprotein C3 (*Apoc3)*, and lipoprotein lipase (*Lpl)* genes in the peroxisome proliferator-activated receptor (PPAR) signaling pathway were important. The regulation of lipid metabolism through the PPAR signaling pathway was important for improving the exercise endurance of rats in our exercise-induced fatigue model. Therefore, we conclude that acupoint catgut embedding can not only promote body fat decomposition and reduce lactic acid accumulation but also promote the repair of tissue damage and liver damage caused by exercise fatigue. Acupoint catgut embedding regulates the PPAR signaling pathway by upregulating *Lpl* expression and downregulating *Slc27a2*, *Fabp1*, and *Apoc3* expression to further improve body fat metabolism.

## 1. Introduction

Exercise-induced fatigue (EF) refers to the inability of the body to sustain power and energy output, resulting in damage to the body’s basic energy-producing mechanisms [1]. EF is, in essence, a nonpathological, multisystem, multifactorial construct with contributions from peripheral muscular and respiratory effects as well as influences from the central nervous system and the psychological state. The accumulation of protons in muscle cells, the depletion of energy, the accumulation of ammonia in blood and tissues, oxidative stress, muscle damage, and changes in neurotransmitter synthesis are some of the main causes of exercise-induced fatigue [2,3,4,5,6]. Both Western allopathic medicine and traditional Chinese medicine have developed approaches to the management of EF. The traditional Chinese medicine approach offers interventions that include various prescriptions [7], acupuncture [8], and other methods to treat EF [9]. Among these, catgut embedding acupuncture was shown to improve EF in human athletes in Yong Zhen Chen’s study. Catgut embedding involves the implantation of an absorbable catgut thread at acupoints to induce prolonged stimulation [10]. Studies treated obese rats fed a high-fat diet with Zusanli and Neiting acupoint treatment once a week for 4 weeks. Catgut-implanted rats were compared with normal rats and unoperated obese rats; catgut embedding was shown to provide long-lasting stimulation of the target acupoints, induce the body’s regulating systems to varying degrees, and help the inherent regulating potential in the body to normalize function. Acupoint embedding has been shown to adjust central and peripheral leptin levels and promotes hypothalamic OB-R gene expression in obese rats to modulate leptin resistance, insulin resistance, and abnormal endocrinology and metabolism [11]. The absorption of organisms through degradable acupuncture threads may enhance immune responses, thereby strengthening the body’s defenses. Electroacupuncture has been shown to relieve postoperative immunosuppression and promote the recovery of immune function in rats by downregulating CD3 T-cell subsets and IL-6 expression levels [12]. In addition, acupoint stimulation can improve the quality of postoperative recovery and enhance the recovery process in patients with radical thyroidectomy [13,14,15]. As such, acupoint catgut embedding provides a novel adjunct therapy for EF, although the mechanisms underlying these effects remain unclear. In this study, we tested how catgut therapy alters the muscle by analyzing gene changes following acupoint catgut embedding in a rat EF model. Our findings provide an important step to understanding how acupuncture catgut embedding improves EF.

## 2. Materials and Methods

### 2.1. Animals and Housing

Clean Sprague-Dawley rats (6 weeks old; male and female) were obtained from the Laboratory Animal Center of the Inner Mongolia Medical University. All animals were housed in a clean controlled-environment animal room at a temperature of 20–22 °C and relative air humidity of 40–60%. Three single-sex rats were housed per cage. Standard chow and drinking water were provided, and bedding was changed daily; drinking water was provided ad libitum. The study was conducted in strict accordance with the "Regulations on the Administration of Laboratory Animals" of the State Council and related policies and regulations [16,17], a protocol approved by the Institutional Animal Care and Use Committee of the Inner Mongolia Agricultural University (License No. SYXK, Inner Mongolia, 2014-0008), and adhered to the Inner Mongolia Agricultural University guidelines.

### 2.2. Experimental Groupings

All experimental animals first underwent 7 days of adaptive feeding. Then, they were exposed to a 3-day habituation period, during which they performed treadmill exercises; any animals that refused to exercise during this time were excluded from the study. All remaining animals were randomly assigned to four groups (n = 6 per group, half male and half female): (1) control group (normal feeding without treadmill training); (2) treadmill group (7 weeks of incremental treadmill exercise); (3) acupoint catgut embedding group 1, which received treatment at acupoints previously identified to be effective by our group [18]: Qiang Feng, Bai Hui., Xie Qi, Han Gou, Yang Wa, and Qian Shen; and (4) acupoint catgut embedding group 2, which received treatment at the same acupoints plus Fei Shu. After the groups were assigned, the appropriate catgut embedding treatment was conducted. The provided acupoint locations were identified based on “Comparative acupuncture and moxibustion”, which is a standard teaching material for agricultural colleges and universities nationwide [19]. Acupoint catgut embedding was carried out per the standards formulated by the National Standards Development Committee of the People’s Republic of China [20]. After catgut embedding was completed, the rats in acupoint catgut embedding group 1 and acupoint catgut embedding group 2 were given a week to recover. During this week, all animals were fed normally, and no treadmill exercise was performed. After the end of the recovery week, the treadmill group, acupoint catgut embedding group 1, and acupoint catgut embedding group 2 commenced the 7-week treadmill exercise period.

### 2.3. Fatigue Model

**Figure 1:**
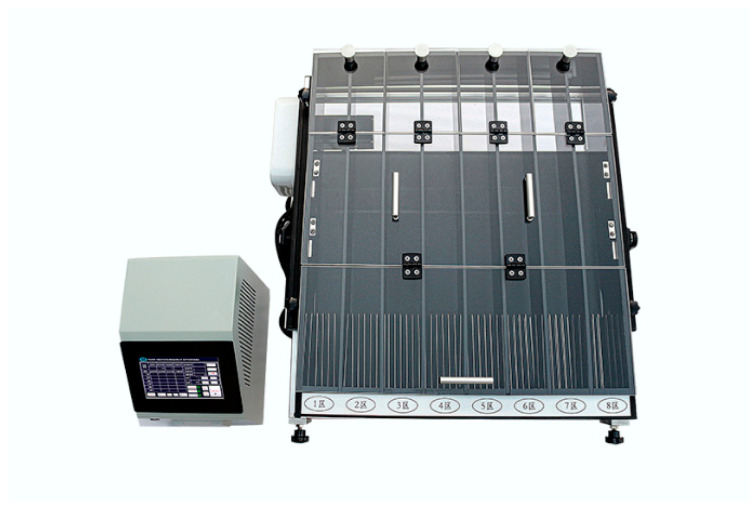
Treadmill setup.

A training treadmill (Zhenghua Biological Instrument Equipment, Huaibei, China) was used during the treadmill exercises; the treadmill setup was as shown in Figure 1. The protocol included 20 min of daily treadmill exercises. The training was conducted Monday through Friday, with rest on Saturday and Sunday, for 7 weeks. In the first 5 weeks, the training velocity was increased weekly by 15, 22, 27, 31, and 35 m per minute (m/min); the training speed was maintained at 35m/min in the 6th and 7th weeks. To encourage the experimental animals to exercise, an electrified metal component was included at the tail of each channel of the treadmill. The stimulus was set to 110 V and 0.5 A. When the experimental animals attempted to leave the treadmill training area, their tail contacted the electrified metal, and an aversive pain stimulus was automatically administered, encouraging a return to treadmill training. On the first day of the 8th week, the animals assigned to the treadmill exercise ran to exhaustion at a speed of 35 m/min. Exhaustion was noted when the animal remained in the last 1/3 of the treadmill for 10 s and was unable to advance after being stimulated. Physical signs of exhaustion following the cessation of running were wheezing, abdominal recumbency, unresponsiveness to electrical stimulation, and the temporary lack of an escape response.

### 2.4. Catgut Embedding Acupoint Treatment

Before thread embedding, a sterile No. 4-0 collagen thread was sectioned (Purchased from Yangzhou ZX Medical Appliance Co., Ltd, Yangzhou, China.) into 3 mm pieces and soaked in sterilized normal saline. The collagen thread was placed in a 7 # thread-embedding needle (0.7 × 65 mm, purchased from Yangzhou ZX Medical Appliance Co., Ltd, Yangzhou, China). The rats were anesthetized by ether aerosol inhalation, and skin around the acupoints was disinfected using a 75 % alcohol solution (Sinopharm Chemical Reagent Co., Ltd, Shanghai, China). With one hand, the thumb and index finger fixed the proposed acupoint, and with the other hand, the needle was used to pierce the acupoint; after reaching the acupoint, the needle core was pushed while the needle tube was withdrawn, and collagen thread was embedded in the subcutaneous tissue and muscle layer of the acupuncture point. After removing the needle, the needle hole was pressed with a sterile dry cotton ball to stop bleeding.

Qiang Feng is located behind the shoulder joint, in the depression formed by the posterior edge of the deltoid muscle, the long head of the arm triceps, and the outer head, and there is one point in each limb. Bai Hui is located in the depression between the last lumbar vertebra and the spinous process of the first sacral vertebra, and there is one point. Fei Shu is located between the penultimate sixth intercostal aces, in the iliocostal groove at the horizontal line of the superior iliac wing, with one point on each side. Xie Qi is located in the biceps femoris muscle sulcus, where it intersects with the horizontal line of the anus. Qian Shen is located in the same muscle sulcus as Xie Qi, about on the horizontal line above the kneecap. Han Gou and Yang Wa are located in the same muscle groove as Xie Qi and Qian Shen, which are between the two acupoints. Both hind limbs have one point of Xie Qi, Han Gou, Yang Wa, and Qian Shen. 

### 2.5. Blood Sample

Blood was collected by cardiac puncture immediately after rat treadmill training to exhaustion. All blood samples were collected by cardiac puncture; the collected blood was coagulated for 2 h at room temperature, 5 mL of blood was centrifuged at 1000× *g* for 10 min at 4 °C to separate the serum, and the serum was stored at −20 °C.

### 2.6. Tissue Samples 

Animals were euthanized following the final session. After sterilizing the anterior thigh, we made an incision in the skin. We identified the m.gluteus superficialis (a triangular lamella, with its posterior origin covered by the anterior head of the biceps femoris, with fascia originating from the dorsal border of the ilium and ending at the third trochanter) and the musculus tensor fasciae latae (a thin fan-shaped muscle on the front of the outer side of the thigh, and its posterior border is fused with the superficial gluteal muscle). After identifying the insertion point, the quadriceps femoris muscle was exposed, and a sample was harvested from it. The quadriceps muscle sample was promptly chilled in a 4 °C precooled diethylpyrocarbonate (DEPC) aqueous solution, which also removed the surface blood and hair. The muscle tissue was then sectioned into approximately 100 g pieces, packed into a cryopreservation tube, and promptly stored in liquid nitrogen.

### 2.7. Gene Analysis

Total RNA was extracted from muscle tissue using Trizol reagent (Invitrogen, Carlsbad, CA, USA). After the total RNA was determined and the purity was assessed (using RSeQC 2.4 software), RNA was purified twice using AMPure XP beads. The purified RNA was reverse-transcribed into cDNA using an mRNASeq sample preparation kit (Illumina, San Diego, CA, USA). End-paired sequencing was performed using Illumina NovaseqTM 4000.

### 2.8. RNA-Seq Analysis

The weighted correlation network analysis (WGCNA) package in R version 3.2.5 was used to analyze the fragments per kilobase of transcript per million fragments mapped (FPKM) value of each transcript. The 20 transcripts with the highest score rates among the six modules were selected for enrichment analysis using the OmicStudio tool (https://www.omicstudio.cn/tool, accessed on 1 May 2021). Differentially expressed genes were selected based on the following criteria: fold change (FC) > 1.5 or FC < 0.667 and *p*-value < 0.05. Gene ontology (GO) enrichment and Kyoto Encyclopedia of Genes and GENOMES (KEGG) enrichment analysis were performed on the identified DEGs.

### 2.9. Mass Spectrometry Metabolomics

To extract metabolites, the muscle sample was mixed with precooled 50% methanol diluted with ultrapure water (D 24 UV, Merck Millipore) and incubated at room temperature for 10 min, and then the mixture was frozen overnight at −20 °C. Then, the mixture was centrifuged at 4000× *g* for 20 min, and the supernatant was collected and stored at −80 °C until LC–MS analysis.

### 2.10. High-Performance Liquid Chromatography and High-Resolution Tandem Mass Spectrometry

HP–LC was conducted using a SCIEX instrument and ACQUITY UPLC T3 columns (100 mm × 2.1 mm, 1.8 µm, Waters, Milford, MA, USA). The mobile phase was divided into liquid A and liquid B; liquid A was composed of 0.1% formic acid and water, and liquid B was composed of 0.1% formic acid and acetonitrile. The injection volume was 4 µL. The flow rate was 0.4 mL/min. The column temperature was 35 °C. The elution mode for gradient elution and the detailed procedures are provided in Table 1.

Mass Spectrometry was conducted using a TripleTOF 5600 plus Triple Quad instrument. The auxiliary and sheath gas pressures were both 60PSI. The ion source was Q-TOF. The positive ion interface voltage was 5 kV, and the negative ion interface voltage was −4.5 kV. The heating module was 650 °C, the scanning mode was IDA, and the acquisition time was 150 ms.

### 2.11. Data Analysis

To determine the effects of exercise, we tested the exercise-only group against the control group. To test the effect of the catgut embedding treatment, we tested the two treatment groups independently against the treadmill group. Group metabolite levels were compared by using a series of Student’s t-tests with corrections for multiple tests via the Benjamini–Hochberg method. Metabolites were subjected to multivariate analysis, mainly using partial least-squares discriminant analysis (PLS-DA), to generate variable importance plots (VIP) for each metabolite. We used the following criteria to identify differentially expressed metabolites: >2–fold difference, q < 0.05, and VIP > 1. The KEGG public database was used to accurately screen and label metabolites, and the differential metabolite pathway was analyzed.

### 2.12. Blood Biochemical Indexes and Detection of Arachidonic Acid and Free Fatty Acids

Serum samples were tested for blood biochemical indices using an automatic blood biochemical (Mindray BS-180 VET) analyzer. We quantified triglyceride (TG), total cholesterol (TC), lactate dehydrogenase (LDH), high-density lipoprotein (HDL), alanine transaminase (ALT), aspartate aminotransferase (AST), and glucose (GLU). The levels of free fatty acids and arachidonic acid in blood were detected using a rat free fatty acid (FFA) ELISA kit (MM-09324R1, Jiangsu Meimian Industrial Co., Ltd, Yancheng, China) and a rat arachidonic acid (ARA) ELISA kit (MM-0929R1, Jiangsu Meimian Industrial Co., Ltd).

### 2.13. Real-Time Quantitative PCR (RT-qPCR)

To validate the transcriptomic data, we performed RT-qPCR on 6 randomly selected differentially expressed genes. In short, total RNA was extracted from tissue samples using a total RNA extraction kit (Tiangen Biotech(Beijing)Co., Ltd, Beijing, China); the concentration of the extracted RNA sample was determined using a nucleic acid quantizer (Eppendorf, Hamburg, Germany), and quality was verified using a 260/280 ratio between 1.8 and 2.0. RNA samples were reverse-transcribed using a Vazyme Reverse transcription kit (HiScriptII Q RT SuperMix for q-PCR, Vazyme biotech Co., Ltd, Nanjing, China) according to the manufacturer’s protocol. The RT-qPCR reaction was carried out using a real-time PCR instrument (Applied Biosystems 7500 Fast, ABI). The primer information is shown in Table 2. Relative changes in gene expression were calculated using the 2-△△CT method. Each sample was tested in triplicate, and group comparisons were tested using a one-way ANOVA in Prism Graph-PAD 6.0 software.

## 3. Results

### 3.1. Genes Altered by Exercise-Induced Fatigue and Treatment

**Figure 2:**
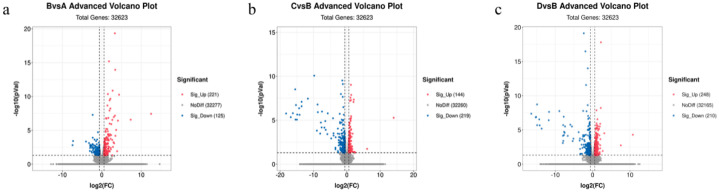
Volcano plot of DEGs among the study comparison groups. (**a**) Volcano plot of DEGs in BvsA. (**b**) Volcano plot of DEGs in CvsB. (**c**) Volcano plot of DEGs in DvsB. A: control group; B: treadmill group; C: acupoint catgut embedding group 1; D: acupoint catgut embedding group 2; DEGs: differentially expressed genes.

We identified 32,623 genes. As shown in the volcano plot (Figure 2), there were 346 differentially expressed genes (DEGs) between the control and treadmill-only groups. These included 221 upregulated DEGs and 125 downregulated DEGs. There were 363 DEGs between the treadmill group and the catgut group 1, which included 144 upregulated DEGs and 219 downregulated DEGs. There were 458 DEGs between the treadmill group and catgut group 2, which included 248 upregulated DEGs and 210 downregulated DEGs.

**Figure 3:**
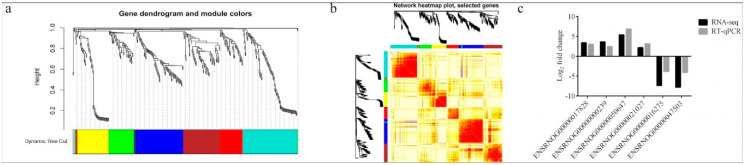
(**a**) WGCNA analysis clustering dendrogram. (**b**) Heatmap of the degree of difference between genes analyzed by WGCNA. (**c**) Comparison of transcriptome and RT-qPCR results. WGCNA: weighted correlation network analysis.

**Figure 4:**
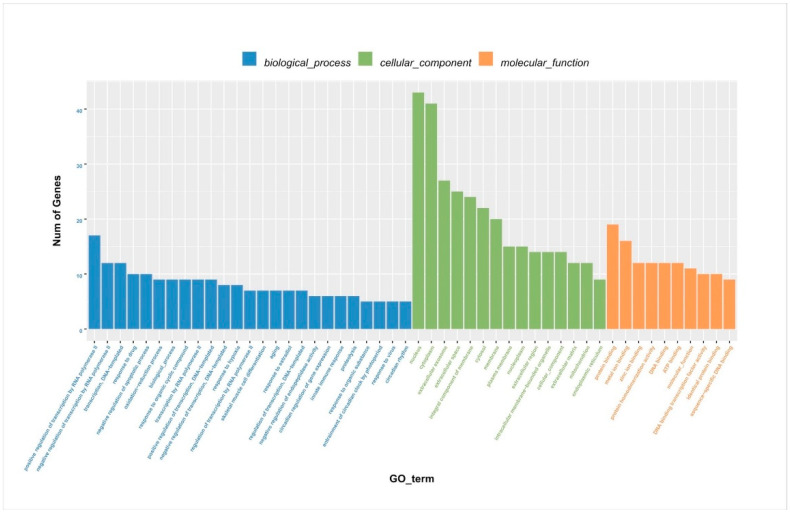
Column chart of DEG GO enrichment analysis. DEGs: differentially expressed genes; GO: gene ontology.

After identifying the DEGs, we analyzed the genes using WGCNA using dynamic and lust functions to merge highly similar modules to obtain a clustering dendrogram. A heatmap was drawn based on the degree of difference between genes. Through the WGCNA analysis clustering dendrogram, the genes were divided into six clusters of correlated genes; the expression levels of common genes in the six modules are well correlated. The top 20 differential genes in the six modules were then subjected to enrichment analysis (Figure 3a,b). Through GO functional annotation, the DEGs were divided into three categories: cellular components, biological processes, and molecular functions. The above three functions were further divided into the positive regulation of transcription by RNA polymerase II, the negative regulation of transcription by RNA polymerase II, participation in nuclear synthesis, synthesis in the cytoplasm, participation in extracellular exosome synthesis, participation in protein binding, participation in metal ion binding, etc. (Figure 4).

**Figure 5:**
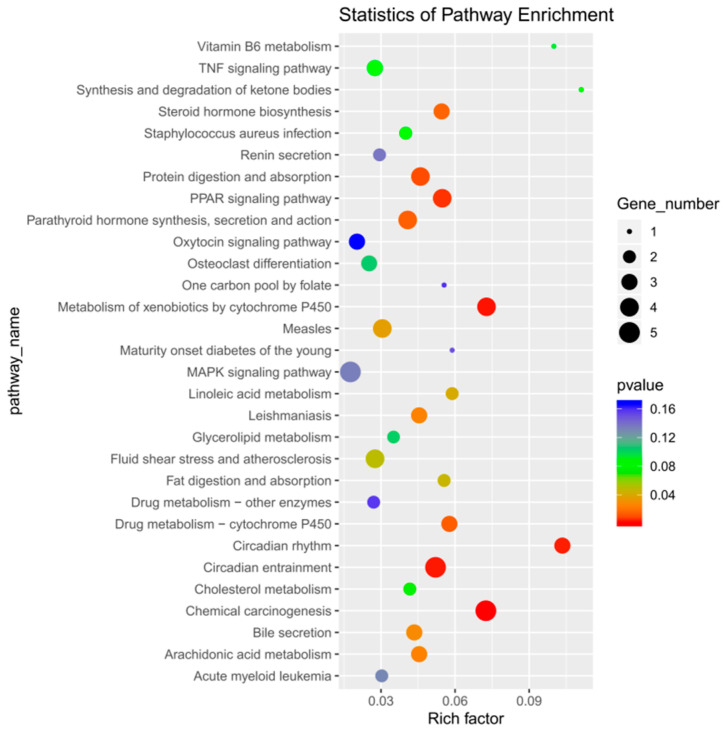
Bubble diagram of DEGs KEGG enrichment analysis. DEGs: differentially expressed genes; KEGG: Kyoto Encyclopedia of Genes and Genomes.

After enrichment analysis, 16 metabolic pathways were found to be significantly enriched (*p* < 0.05), which included chemical carcinogenesis, metabolism of cytochrome P450 to exogenous substances, circadian entrainment, circadian rhythm, protein digestion and absorption, peroxisome proliferator-activated receptor (PPAR) signaling pathways, drug metabolism–cytochrome P450, parathyroid hormone synthesis, secretion, and action, steroid hormone biosynthesis, leishmaniasis, arachidonic acid metabolism, bile secretion, measles, linoleic acid metabolism, fat digestion and absorption, and fluid shear stress and atherosclerosis. By inspecting the bubble enrichment plot, we decided that the following would be important targets of our future studies: protein digestion and absorption; PPAR signaling pathway; synthesis, secretion, and action of parathyroid hormone; and other metabolic pathways (Figure 5). Between the treadmill and control groups, genes ENSRNOG00000017828 and ENSRNOG00000000239 were elevated. Between acupoint catgut embedding group 1 and the control group, gene ENSRNOG00000050647 was elevated. Between acupoint catgut embedding group 1 and the treadmill group, genes ENSRNOG00000021027 and ENSRNOG00000016275 were elevated. Between acupoint catgut embedding group 2 and the treadmill group, gene ENSRNOG00000047503 was downregulated. These expression trends were consistent with our transcriptomic data. The RT-qPCR verification results were consistent with the RNA sequencing data, showing that the transcriptome RNA-seq data were reliable (Figure 3c).

### 3.2. Metabolites Altered by Exercise-Induced Fatigue and Treatment

**Figure 6:**
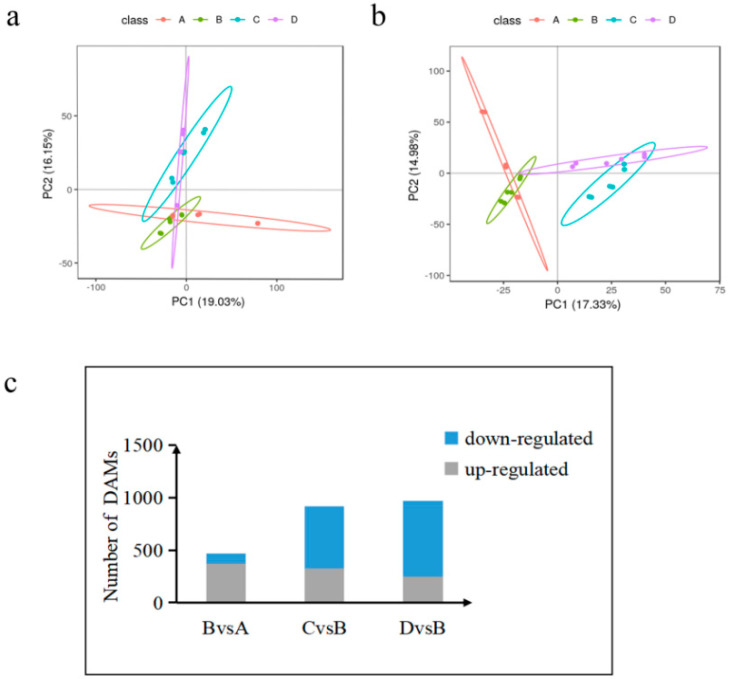
Accumulated metabolite PLS-DA and analysis of DAMs in each comparison group. (**a**) Core diagram of PLS-DA model for each comparison group in the case of positive model. (**b**) Core diagram of the PLS-DA model for each comparison group in the case of negative model. (**c**) Differential number of DAMs in each comparison group. A: control group; B: treadmill group; C: acupoint catgut embedding group 1; D: acupoint catgut embedding group 2. PLS-DA: partial least-squares discriminant analysis; DAMs: differentially accumulated metabolites.

We conducted a principal component analysis (PCA) and constructed a PCA map of metabolites by PLS-DA modeling for the screened ions, with the fractions of PC1 and PC2 as its horizontal and vertical coordinates, as shown in Figure 6a,b. The metabolite ions were well separated at each time point, indicating that the model is reliable and stable. It shows that the positive/negative ion analysis system is relatively stable during the analysis of this sample in each group, and the obtained analysis data can generally reflect the real state of the sample.

We identified 11,588 metabolites. As shown in Figure 3c, 470 differentially accumulated metabolites (DAMs) were identified in the treadmill group versus the control group, accounting for 4.05% of the total detected metabolites. There were 920 DAMs identified between the treadmill group and catgut group 1, accounting for 7.94% of the total detected metabolites, and 972 DAMs were identified between the treadmill group and catgut group 2, accounting for 8.39% of the total detected metabolites. We then generated a Venn diagram to display these DAMs (Figure 6c). By visual inspection, 144 DAMs were jointly enriched in treadmill vs. control, treadmill vs. catgut 1, and treadmill vs. catgut 2 groups. Additionally, 144 DAMs were jointly enriched in treadmill vs. control and treadmill vs. catgut 1 groups. There were 59 DAMs in the treadmill vs. control and treadmill vs. catgut 2 groups and 270 DAMs in the treadmill vs. catgut 1 and treadmill vs. catgut 2 groups.

**Figure 7:**
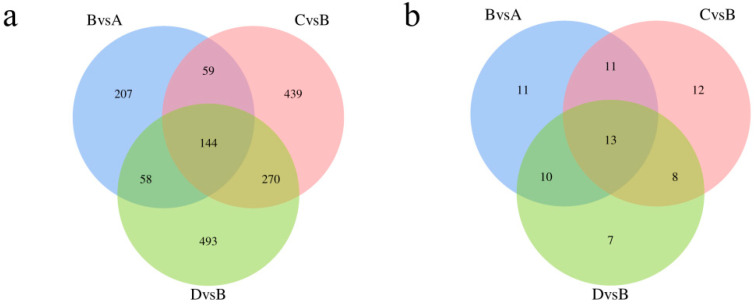
Venn diagram of DAMs and KEGG enrichment analysis. (**a**) Venn diagram of DAM enrichment analysis between each comparison group. (**b**) Wayne diagram of enrichment signaling pathway in each comparison group. A: control group; B: treadmill group; C: acupoint catgut embedding group 1; D: acupoint catgut embedding group 2. DAMs: differentially accumulated metabolites; KEGG: Kyoto Encyclopedia of Genes and Genomes.

KEGG pathway enrichment found a number of unique signaling pathways that differentiated the experimental groups (Figure 7a). Table 3 shows the 13 signaling pathways that were jointly enriched in the three comparison groups. Analyzing the signaling pathways identified by transcriptomic and metabolomic analyses, we can see in Figure 7b that there are seven signaling pathways identified by both approaches; these are shown in Table 4. There were 120 signaling pathways enriched by RNA-seq and 6 signaling pathways enriched by metabolomics alone. After a combined analysis of the transcriptome and metabolome, we focused on *Slc27a2*, *Fabp1*, *Apoc3*, and *Lpl* genes in the PPAR signaling pathway.

### 3.3. TG, TC, LDH, HDL, ALT, AST, GLU, Arachidonic Acid, and Free Fatty Acid in the Blood Altered by Exercise-Induced Fatigue and Treatment

**Figure 8:**
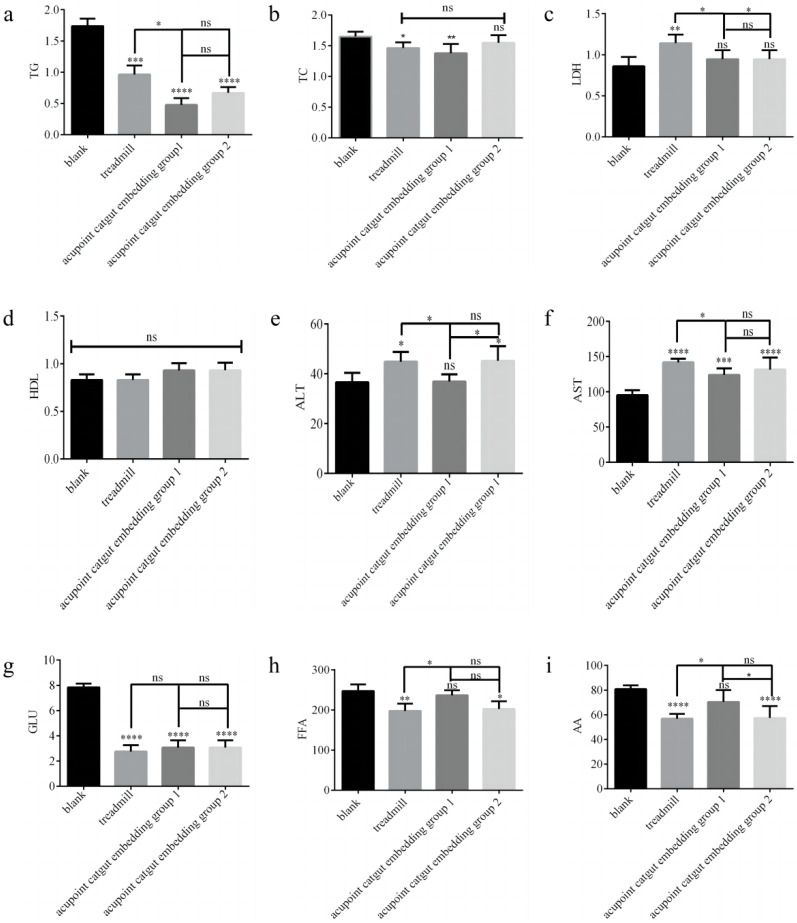
Biomarkers of lipid metabolism in the blood. (**a**) TG, (**b**) TC, (**c**) LDH, (**d**) HDL, (**e**) ALT, (**f**) AST, (**g**) GLU, (**h**) FFA, and (**i**) AA. * *p* < 0.05, ** *p* < 0.01, *** *p* < 0.001, **** *p* < 0.0001; ns: not significant (*p* > 0.05). TG: triglyceride; TC: total cholesterol; LDH: lactate dehydrogenase; HDL: high-density lipoprotein; ALT: alanine transaminase; AST: aspartate aminotransferase; GLU: glucose; AA: arachidonic acid; and FFA: free fatty acid.

The TG, TC, LDH, ALT, AST, and GLU levels were measured by using a fully automated blood biochemical analyzer, as shown in Figure 8a–g. Serum FFA and AA were determined using ELISA assay kits, and the results are shown in Fig. 8h-i. Compared with the control group, in the treadmill group, LDH, ALT, and AST were significantly higher (*p* < 0.05), and TG, TC, GLU, FFA, and AA were significantly lower (*p* < 0.05); in acupoint catgut group 1, AST was significantly higher (*p* < 0.05), and TG, TC, and GLU were significantly lower (*p* < 0.05); and in acupoint catgut group 2, ALT and AST were significantly higher (*p* < 0.05), and TG, GLU, FFA, and AA were significantly lower (*p* < 0.05). Compared with the treadmill group, TG, LDH, ALT, and AST were significantly lower in acupoint catgut embedding group 1 (*p* < 0.05); FFA and AA were significantly higher in acupoint catgut embedding group 1 (*p* < 0.05); and LDH was lower in acupoint catgut embedding group 2 (*p* < 0.05). Compared with acupoint catgut embedding group 1, acupoint catgut group 2’s ALT was significantly higher (*p* < 0.05), and AA was significantly lower (*p* < 0.05). There were no other significant between-group differences.

### 3.4. Effect of Acupoint Catgut Embedding on the PPAR Signaling Pathway

We next scrutinized the effect of catgut embedding on the four targets. *Slc27a2*, also known as FATP2, plays a role in the transport of long-chain fatty acids across the cell membrane and their intracellular accumulation [21]. Fatty acid binding protein 1 (*Fabp1*) has a wide range of ligands, including fatty acyl-CoA, bilirubin, heme, hydroxyl and hydroperoxide metabolites of fatty acids, other hydrophobic ligands, and other substances, indicating that *Fabp1* has a variety of functions and effects [22]. *Apoc3* is also a key factor in plasma triglyceride metabolism, as are other apolipoproteins [23]. *Lpl* plays a key role in lipid metabolism by catalyzing intravascular triglyceride hydrolysis to fatty acids by catalyzing proteins such as lipoproteins [24].

**Figure 9:**
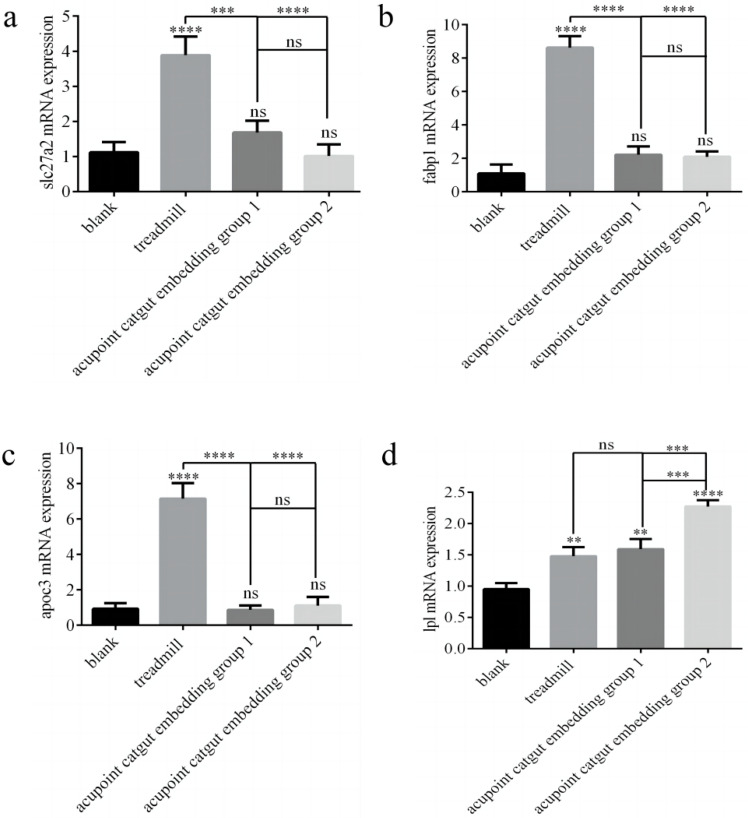
*Slc27a2*, *Fabp1*, *Apoc3*, and *Lpl* gene mRNA and protein expression in the quadriceps femoris related to the PPAR signaling pathway. (**a**) *Slc27a2* mRNA, (**b**) *Fabp1* mRNA, (**c**) *Apoc3* mRNA, and (**d**) *Lpl* mRNA. ** *p* < 0.01, *** *p* < 0.001, **** *p* < 0.0001; ns: not significant (*p* >0.05). *Slc27a2*: solute carrier family 27 member 2; *Fabp1*: fatty acid binding protein 1; *Apoc3*: apolipoprotein C3; *Lpl*: lipoprotein lipase.

The expression of *Slc27a2*, *Fabp1*, *Apoc3*, and *Lpl* genes was determined by RT-PCR. Compared with controls, the treadmill group showed greater expression of *Slc27a2*, *Fabp1*, and *Apoc3* mRNA (*p* < 0.05), and there were no significant differences among the other groups (*p* > 0.05) (Figure 9a–c). Compared with the model group, catgut 1 and catgut 2 groups showed lower *Slc27a2* mRNA (*p* < 0.05), and the differences between the other groups were not significant. Compared with the control group, the model, catgut 1, and catgut 2 groups showed higher expression of *Lpl* mRNA (*p* < 0.05) (Figure 9d). In addition, *Lpl* mRNA in the catgut 2 group was higher than in the model group (*p* < 0.05). Differences in *Lpl* mRNA expression between the other groups were not significant (*p* > 0.05).

## 4. Discussion

Around 60 years ago, it was established that fat is transported to muscle cells in the form of free fatty acids (FFAs) [25]. The ability to transport, ingest, and oxidize fatty acids is critical to meeting the metabolic demands of endurance exercise, as carbohydrates do not solely meet the energy demands of prolonged exercise. The lipolysis of fat is stimulated by exercise-induced catecholamine hormones, and the oxidation of fatty acids from lipolysis helps avert the depletion of glycogen stores by providing fuel for prolonged exercise [26,27]. 

Luiz Gustavo Marin Emed measured acute changes in lipid levels in ultramarathon runners who completed an event and found that there was a downward trend in TG and TC without changes in HDL, indicating that exercise can reduce triglycerides and cholesterol due to their mobilization from visceral and subcutaneous adipose tissue, and that VLDL-C in TG is broken down into FFA by lipase. Elevated LDH demonstrated an increase in muscle injury following endurance exercise [28]. In our study, TG and TC were lower in the treadmill group compared to the control group, and TG was lower in acupoint embedding group 1 than in the treadmill group. These findings show that EF promoted fat oxidation and decomposition, that TG and TC were reduced by EF, and that acupoint embedding can promote adipolysis. No obvious differences were seen in TG and TC levels in acupoint embedding group 2 compared to the treadmill group, indicating that there was no obvious effect on adipolysis in rats in acupoint embedding group 2. Serum LDH was increased by exercise fatigue but reduced in the catgut groups; there was no significant difference in LDH between the two catgut groups. These data show that catgut embedding can alleviate exercise-induced fatigue by reducing blood LDH, which may reduce the amount of lactate accumulation in skeletal muscle.

Sprague-Dawley rats swim-trained for one hour daily for one month show increased serum AST and ALT, demonstrating that intense exercise can induce liver stress and injury [29]. We found that our treadmill group showed elevated ALT and AST compared to controls, corroborating these results. Catgut group 1 showed reduced ALT and AST, demonstrating that this treatment can promote the repair of liver injury due to EF; catgut group 2 did not show this reduction, suggesting that the specific acupoints utilized in the former group are required for the effect.

Six weeks of intensive endurance training (10 men and 3 women aged 29 ± 2) was previously shown to reduce plasma free fatty acid levels [30]. Dogs weighing 10–15 kg were pancreatectomized under morphine-pentothal anesthesia. Sodium-L(+)lactate (pH 7.4) was infused intravenously; as the blood lactate level rose, plasma FFAs decreased, showing a negative correlation between the plasma FFA level and the concentration of blood lactate [31]. In this study, we also found that free fatty acids were reduced in the treadmill group compared to the control group; this reduction may be due to reduced muscle glycogen, increased lactate production and lactic acid accumulation, and reduced free fatty acids. Catgut embedding group 1 significantly increased serum free fatty acids, and their levels were not different from those in the control group. This shows that catgut treatment attenuates the effects of exercise-induced fatigue.

Fourteen individuals who frequently exercised (six men and eight women, age 36.9 ± 8.4) were provided additional exercise for 6 weeks; blood AA was shown to decrease with the increasing intensity of exercise. This demonstrated an increase in fatty acid metabolism and utilization by increasing skeletal muscle blood flow during exercise [32]. Serum cytokines were measured in 10 male subjects (mean age 27.5 years, range 24–37 years) after a marathon, showing that strenuous exercise can induce an increase in proinflammatory and inflammation-responsive cytokines [33]. Serum fatty acids and cytokines were tested in blood samples from 595 men and 748 women in the Chianti area, showing that serum AA is inversely associated with blood inflammatory markers [34]. In this study, we also found reduced AA in the treadmill group compared to the control group. In catgut embedding group 1, serum AA significantly increased to similar levels to the control group. This shows that catgut treatment can mitigate the inflammatory response to exercise.

In one study, obese individuals engaged in at least three weekly aerobic exercise sessions and ate a low-calorie diet for three months. FATP2, also known as *Slc27a2*, was found to modulate the obesity index and cardiorespiratory fitness in this group [35]. We also found that the expression of the fatty acid transporter *Slc27a2* was increased in the treadmill group compared to the control group. In the catgut group, *Slc27a2* was reduced, which may suggest the normalization of FFA mechanisms.

*Fabp1* plays a central role in the transport and utilization of fatty acids in cells. Besides participating in the uptake, transport, and metabolism of lipid ligands such as long-chain fatty acids, *Fabp1* also participates in the regulation of mitosis, cell growth, and differentiation [36,37,38]. Various animal and cell-based experimental models have shown that *Fabp1* plays a crucial role in regulating liver lipid metabolism. At the same time, studies have shown that the downregulation of *Fabp1* helps to enhance the clearance of autophagy [39]. In one study, C57BL/6 mice exercised for 12 weeks while receiving a high-fat diet. Exercise was found to be protective against the adverse hepatic effects of the diet; the effects were thought to be mediated by a reduction in *Fabp1*, resulting in improved hepatic lysosomal activity [40]. In our study, we found that *Fabp1* was increased in the model group, which may be related to disordered lipid metabolism due to exercise fatigue. This would result in the increased uptake and accumulation of fatty acids in target tissues and impaired cell lysosome function, which in turn enhances autophagy. Compared with the model group, *Fabp1* was downregulated in the catgut embedding group, which suggests that this treatment was able to restore lysosomal function, which would help to enhance autophagic clearance and reduce lipid accumulation in the muscle.

We also found that *Apoc3* was increased in the treadmill group compared with the control group, which may be due to exercise fatigue leading to impaired lipid metabolism. Compared with the treadmill group, *Apoc3* was downregulated after the acupoint catgut embedding pretreatment, indicating that acupoint catgut embedding normalized lipid metabolism. Studies have shown that *Apoc3* inhibits *Lpl*-dependent plasma triglycerides, and clearance stimulates hepatic VLDL secretion [41,42]. The utilization of plasma triglycerides depends on the disturbance of lipid metabolism in the body caused by exercise-induced fatigue. The upregulated expression of *Lpl* in the model group may be due to insufficient energy supplies caused by exercise fatigue, which promotes the upregulation of *Lpl* expression and further catalyzes the hydrolysis of triglycerides in plasma to fatty acids. Compared with the model group, the expression of *Lpl* was upregulated after the acupoint catgut embedding pretreatment, indicating that acupoint catgut embedding may promote the hydrolysis of triglycerides in the body’s plasma to fatty acids by upregulating the expression of *Lpl*, and the fatty acids are further oxidized to provide energy. In other studies, *Apoc3* was found to inhibit *Lpl*-dependent plasma TG clearance; this is consistent with our findings [43].

## 5. Conclusions

We found that acupoint catgut embedding can promote fat decomposition, reduce lactic acid accumulation, and also promote tissue damage repair and liver damage repair caused by exercise fatigue. Catgut embedding acupuncture improves rat exercise tolerance by regulating lipid metabolism through the PPAR signaling pathway. This involves the upregulation of *Lpl* and the downregulation of *Slc27a2*, *Fabp1*, and *Apoc3* to enhance fat metabolism and thereby energy availability.

## Figures and Tables

**Table 1:** HP-LC gradient elution parameters.

Time/min	Liquid B Composition in the Mobile Phase
0–0.5 min	5 B%
0.6–7 min	5–100% B
7–8 min	100% B
8–8.1 min	100–5% B
8.1–10 min	5% B

**Table 2:** Primer sequences.

Primer Name	Forward (5′–3′)	Reverse (5′–3′)
ENSRNOG00000017828	TGTGCCAGGACAACATCATTAGC	TAGCCGTGGTCGTCTGCGTAC
ENSRNOG00000000239	AGATGAACAGAAACCAACCC	AGTCCACCCATTTCAGCACAGTTC
ENSRNOG00000050647	CAGGTGAACTACAAGGGCGAGAAC	AGTAGGCGGGCACGGTGATC
ENSRNOG00000021027	CACCGCTTCTCAGAGGAGGAATTG	TCGACCTCTTGGCTGCTTCATTG
ENSRNOG00000016275	GTCAGATTGGCAGGGATCAGCAG	ATATCAGTCCAGCGAGGCAGAGG
ENSRNOG00000047503	TACTGGAGCAAGTTCACTGATAAG	GGAGGCAGCAGGATAGATGG
*Slc27a2*	TACTGGAGCAAGTTCACTGATAAG	GTAGCAGAGACTTGGCACGGATG
*Fabp1*	CCAGAAAGGGAAGGACATCAAGGG	TGGTCTCCAGTTCGCACTCCTC
*Apoc3*	GCCCTGAGGACCAACTAACAACAC	TTCGGAGGCAGCAGGATAGATGG
*Lpl*	GATTTACACGGAGGTGGACATCGG	ACCAGTCTGACCAGCGGAAGTAG
*β-Actin*	CTGAGAGGGAAATCGTGCGTGAC	AGGAAGAGGATGCGGCAGTGG

**Table 3:** Common signaling pathways in three comparison groups.

Pathway ID	Pathway Name
map01210	2-Oxocarboxylic acid metabolism
map00140	Steroid hormone biosynthesis
map01523	Antifolate resistance
map04913	Ovarian steroidogenesis
map05200	Pathways in cancer
map05215	Prostate cancer
map04726	Serotonergic synapse
map00590	Arachidonic acid metabolism
map03320	PPAR signaling pathway
map00061	Fatty acid biosynthesis
map00071	Fatty acid degradation
map01040	Biosynthesis of unsaturated fatty acids
map00591	Linoleic acid metabolism

**Table 4:** Signaling pathways enriched by transcriptomics and metabolomics.

Pathway ID	Pathway Name
map00140	Steroid hormone biosynthesis
map01523	Antifolate resistance
map05200	Pathways in cancer
map04726	Serotonergic synapse
map00590	Arachidonic acid metabolism
map03320	PPAR signaling pathway
map00591	Linoleic acid metabolism

## Data Availability

All raw data generated in this project has been deposited to the gene expression omnibus (GEO) with the accession code GSE220191.
